# Implementing Contralateral Surgical Exploration during Hernia Repair in Children with Unilateral Inguinal Hernia: A Dutch Qualitative Study

**DOI:** 10.3390/children10101631

**Published:** 2023-09-29

**Authors:** Kelly M. A. Dreuning, Femke Van Nassau, Johannes R. Anema, L. W. Ernest Van Heurn, Joep P. M. Derikx

**Affiliations:** 1 Department of Pediatric Surgery, Emma Children’s Hospital, Amsterdam UMC, University of Amsterdam & Vrije Universiteit Amsterdam, Amsterdam Reproduction and Development Research Institute and the Amsterdam Public Health Research Institute, Meibergdreef 9, 1105 AZ Amsterdam, The Netherlands; k.m.dreuning@amsterdamumc.nl (K.M.A.D.);; 2 Department of Public and Occupational Health, Amsterdam Public Health Institute, Amsterdam University Medical Centers, Vrije Universiteit Amsterdam, De Boelelaan 1117, 1081 HV Amsterdam, The Netherlandsh.anema@amsterdamumc.nl (J.R.A.)

**Keywords:** inguinal hernia, inguinal hernia repair, contralateral exploration, metachronous hernia, qualitative research

## Abstract

A total of 10–15% of children undergoing unilateral inguinal hernia repair develop a metachronous contralateral inguinal hernia (MCIH) that necessitates second anesthesia and surgery. Contralateral exploration can be performed to prevent MCIH development. This study investigates (1) factors that promote or hinder the adoption and (de-)implementation of contralateral groin exploration in children ≤ 6 months undergoing unilateral hernia repair and (2) strategies to overcome these barriers. A qualitative interview study was conducted using 14 semi-structured interviews and two focus groups involving healthcare professionals, stakeholders involved from a patients’ perspective and stakeholders at the organizational/policy level. The results show that the effectiveness of surgical treatment and stakeholders’ motivation and attitudes towards the intervention were reported as barriers for implementation, whereas patient and family outcomes and experience and strategies to overcome these barriers were identified as facilitating factors for future implementation. This study is unique in its contributions towards insights into facilitators and barriers for (de-)implementation of contralateral groin exploration in children with a unilateral inguinal hernia. In case the HERNIIA trial shows that contralateral exploration is beneficial for specific patient and family outcomes or a subgroup of children, the results of this study can help in the decision-making process as to whether contralateral exploration should be performed or not.

## 1. Introduction

Inguinal hernia repair is one of the most commonly performed surgeries in children. In 80% of patients, the hernia is unilateral at the time of presentation, but after surgical repair, 10–15% of the children develop a hernia on the contralateral side: a metachronous contralateral inguinal hernia (MCIH) necessitating second surgery [1]. Infants younger than six months have the highest risk of developing an MCIH [1,2]. Preventive strategies for the development of an MCIH have been proposed since the 1950s. It has been reported that in 40% of children with a unilateral inguinal hernia, there is a contralateral patent processus vaginalis (PPV) and that exploring the asymptomatic contralateral groin and closing a PPV, if present, can prevent the development of an MCIH and subsequent second anesthesia and surgery [3]. It is estimated that nine contralateral explorations would be required to prevent one MCIH in children under the age of six months [1,2]. However, as contralateral exploration carries risks and not all PPVs develop into clinically relevant hernias, this may be regarded as an unnecessary surgical risk [4].

A survey on the variability of inguinal hernia repair techniques in 2002 showed that 40% of pediatric surgeons would routinely explore the contralateral side in boys younger than two years, 39% in girls younger than five years and 51% would explore the contralateral groin in a premature infant [5]. In 2005, these results were confirmed and showed that 27% of pediatric surgeons would routinely explore the contralateral side, 12% would never consider it, and pediatric surgeons practicing outside of the US were less likely to perform routine contralateral exploration [6]. Nowadays, the evidence comparing contralateral exploration to unilateral repair without contralateral exploration is still sparse. The recently published European guideline on the surgical management of pediatric inguinal hernias still states that no clear recommendation can be made regarding whether contralateral exploration should be performed at the time of open unilateral inguinal hernia repair or not [7].

Therefore, in 2018, the Hernia Exploration oR Not In Infants Analysis (HERNIIA) trial was initiated. This trial is the first randomized controlled trial (RCT) that studies which treatment strategy is more (cost-)effective in infants aged six months or younger with a unilateral inguinal hernia: unilateral hernia repair with or without contralateral exploration [8]. Successful (de-)implementation of the best treatment strategy arising from the results of the HERNIIA trial—in case contralateral exploration is beneficial for specific patient and family outcomes or a subgroup of children—depends on the deployment of implementation strategies that match experienced facilitators and barriers by stakeholders involved in the treatment. Yet, little is known about barriers to and facilitators of the implementation of routine contralateral exploration in pediatric clinical practice. This study therefore aims to identify (1) factors that promote or hinder the adoption and (de-)implementation of contralateral exploration aimed at reducing second surgery because of MCIH development in children aged six months or younger undergoing unilateral hernia repair and (2) strategies to overcome these barriers.

## 2. Materials and Methods

### 2.1. Study Design

This exploratory qualitative study was conducted parallel to our multicenter RCT comparing the effectiveness of groin exploration during unilateral hernia repair (HERNIIA trial) [8]. Fourteen semi-structured interviews and two focus groups were held with relevant stakeholders to explore the potential barriers to and facilitators of the implementation of contralateral exploration. Reporting was performed in accordance with the Consolidated criteria for reporting qualitative studies (COREQ) guidelines [9]. 

### 2.2. Study Population

Different stakeholders from a wide perspective were included in this study: (a) healthcare professionals, e.g., (pediatric) surgeons, surgical residents, anesthesiologists and pediatricians; (b) stakeholders involved from a patients’ perspective, i.e., parents of patients and the Child and Hospital Foundation; and (c) stakeholders at the organizational/policy level, i.e., surgeons with leading positions in the Dutch College of (Pediatric) Surgeons. To promote heterogeneity among participants, we started by reaching out to participants involved in the trial, but we also used a snowball sampling technique to further include stakeholders mentioned by interviewees and determined what stakeholders (e.g., healthcare insurers) did not need to be interviewed. Surgeons and surgical residents were invited from both university and non-university hospitals and selected based on their reluctance or support towards contralateral exploration. Parents of children who chose to participate in the HERNIIA trial, as well as parents who rejected participation, were included to obtain a variety of perspectives. Heterogeneity was ensured by including parents of children who underwent unilateral hernia repair alone (i.e., unilateral group), unilateral hernia repair with simultaneous contralateral exploration (i.e., contralateral exploration group) and initial unilateral hernia repair with the subsequent development of a metachronous contralateral hernia requiring second surgery (i.e., MCIH group). The final number of interviews was determined when data saturation (i.e., the point when no new information was discovered in data analysis) was reached. 

### 2.3. Data Collection and Analysis

To explore potential barriers to and facilitators of the implementation of contralateral exploration, a topic guide was developed in close collaboration with qualitative research and implementation experts and key stakeholders (available upon request). It was based on the innovation framework developed by Fleuren et al., which describes determinants of innovations in healthcare that influence its adoption, implementation and maintenance, according to different categories, i.e., the socio-political context (including parents’ perspective), the organizational context, factors concerning healthcare professionals and determinants related to the intervention itself [10,11]. If necessary, the topic list included specific questions per stakeholder group and was refined after each interview. All interviews were performed using ZOOM or Microsoft Teams due to the COVID-19 pandemic. Interviews were performed by two experienced researchers in qualitative interviewing (KD, FvN). The focus groups were led by a trained and independent ‘neutral’ moderator. All interviews, including focus groups, were audio-recorded and transcribed verbatim. 

Barriers and facilitating factors were identified by performing thematic analysis [12]. The first seven transcripts were open-coded by two researchers (KD and FvN) individually. The two researchers then reached consensus on codes through discussion, and subsequently clustered them into overarching codes and agreed upon the final presentation of the coding book. The codes were categorized into the domains of the framework of Fleuren et al., and all transcripts were subsequently coded using the developed codebook (available upon request) [10]. Upon analysis of the existing codes together with their corresponding quotations, the codes were sorted and subdivided into overarching themes and subthemes. Coding was performed using the Atlas.ti [ATLAS.ti Scientific Software Development GmbH, Berlin, Germany] (version 8.2.24) software package for qualitative data analyses. 

## 3. Results

Twenty-six individuals representing three stakeholder groups participated in the present study. We organized two focus groups, including twelve parents of nine children who were participating in the HERNIIA study. Five interviews were held with parents of children who declined participation in the HERNIIA study. Nine semi-structured interviews were conducted with healthcare professionals engaged in the care of children with an inguinal hernia. Demographic characteristics of the participants are shown in Table 1 and Table 2.

Upon analyzing the data, three themes with subsequent subthemes were identified, as listed in Table 3.

### 3.1. Theme 1: Effectiveness of Surgical Treatment

#### 3.1.1. Available Evidence

Many of the interviewed stakeholders reported that currently available evidence to perform contralateral exploration is very limited, inconclusive and one of the key barriers for the implementation of contralateral exploration: “*In dubio abstine*, *i.e.*, *in case of doubt*, *don’t do”—Pediatric surgeon.* This was mainly mentioned because over 80% of patients will never develop an MCIH, and contralateral exploration and its subsequent risks would thus be unnecessary. *“You should not do anything extra in case it is not necessary. Why should you fix something that isn’t broken?”—Pediatrician.* In the majority of patients, surgical exploration of the contralateral side either shows a closed processus vaginalis (i.e., a negative contralateral exploration), or a patent processus that will never actually develop into a clinically relevant hernia. As one pediatric surgeon explained: “*In that case*, *we might also already remove the appendix and pierce their ears and belly button*, *just in case they want to wear piercings when they are 16 years old*”. In addition, interviewees mentioned that routine contralateral exploration potentially yields additional (unnecessary) complications by expanding anesthetic exposure and performing a second ‘preventative’ intervention on a former healthy side: “*The less you have to operate the better. Here’s the trade-off that you have to perform an extra surgery without knowing whether the patient will actually benefit from it”—Pediatrician–neonatologist.*

According to interviewed healthcare physicians and parents of children who declined participation in the HERNIIA trial, the subsequent benefits of preventing second anesthesia and surgery in only 10–15% of the children who actually develop a clinically relevant (i.e., symptomatic) hernia on the contralateral side did not outweigh these potential (unnecessary) complications. They mainly concluded this based on the “*do no harm principle; don’t do anything in case it is not necessary”*, as pointed out by one of the surgeons. One of the pediatrician–neonatologists stated: “*It is at the expense of something else. If second surgery could be prevented without any risks or sacrifices*, *everyone would do it*”. From an anesthetic point of view, contralateral exploration could be preferred in case it shortens total anesthesia time. However, anesthesiologists also reported that anesthesia should not be prolonged for a procedure that is not indicated at that moment. Moreover, there is no contra-indication for a later, second exposure to anesthetics in case a metachronous hernia develops.

When we asked about the ‘threshold number’ for the effectiveness of contralateral exploration, interviewees suggested that in case the risk for MCIH development in a specific group of children increases to, e.g., between 20 and 50%, routine contralateral exploration should be taken into reconsideration. One of the surgeons mentioned that in preterm born infants, the threshold to deliberate on performing contralateral exploration might be even lower (30% instead of 50%) as they have a higher risk of developing an MCIH. Increasing evidence to perform contralateral exploration was mentioned to facilitate its implementation. 

#### 3.1.2. Technical Aspects and Different Types of Anesthesia and Surgery

Surgeons identified the upcoming use of spinal anesthesia as a potential barrier for implementing contralateral exploration. First, the potential neurological (long-term) effects following spinal anesthesia are expected to be less compared to general anesthesia, which weakens the importance of avoiding second anesthesia. Surgeons also believe spinal anesthesia limits the total operating time to a maximum of 50–60 min, in which unilateral hernia repair with contralateral exploration is not always possible. According to anesthesiologists, the morbidity and mortality following both spinal and general anesthesia in children is almost negligible since it is always performed by experienced pediatric anesthesiologists. They consider it therefore not necessary to prevent second anesthesia in case an MCIH develops and believe that surgery should never be adapted to the type of anesthesia. “*I believe that we fit the type of anesthesia to the type of surgery that is considered necessary. In our experience it is possible to perform bilateral hernia repair using spinal anesthesia. Contralateral exploration does therefore not on forehand rule out the use of spinal anesthesia”—Anesthesiologist.* Though spinal anesthesia does potentially have less side effects in children, not many anesthesiologists expressed familiarity with this type of anesthesia.

Difficulties during anesthesia appeared to be both a barrier and facilitator. Interviewees mentioned that in some children (e.g., neonates), it is preferred to shorten surgical time as much as possible, while in others, it is better to directly explore the other side to prevent future second anesthesia. Especially in cases in which the anesthesiologist experiences difficulties or (premature) patients suffer from complications during anesthesia, both anesthesiologists and pediatricians advocate to perform contralateral exploration simultaneous to unilateral hernia repair to prevent second surgery and especially second anesthesia with potentially the same difficulties.

Laparoscopic repair in the Netherlands is currently only recommended in children with an ipsilateral recurrent hernia [13]. Surgeons who were interviewed mention that the introduction of laparoscopic hernia repair in children with a unilateral hernia facilitates the implementation of contralateral exploration. However, they preferred open inguinal hernia repair since surgeons lack competence and there is an increased risk of complications following laparoscopic repair. One surgeon about his vision on laparoscopic hernia repair in children: “*I believe the cure is worse than the disease*”. 

#### 3.1.3. Complications of Anesthesia and Surgery 

Many healthcare professionals stated that inguinal hernia repair in itself is a relatively ‘minor’ procedure that is performed via a small incision accompanied by few complications. It is only performed by anesthesiologists and surgeons with expertise in anesthetizing and operating on children, and the risk of complications decreases with increasing experience of the treating surgeon: *“It is mainly about the experience and competence in treating inguinal hernia repair; by preventing damage to the spermatic cord and vessels in boys*, *and the detection and ligation of the hernia sac”—Pediatric surgeon.* However, potential (long-term) complications following contralateral exploration (e.g., the increased risk of testicular atrophy in case of a postoperative swelling) were also repeatedly reported to hinder implementation. Surgeons with long-term expertise in treating pediatric inguinal hernias mentioned that in contralateral exploration, it is sometimes hard to detect whether patent processus vaginalis exists or not and that the intervention itself to look for it can sometimes cause more damage (e.g., prolonged anesthesia and long-term problems, such as testicular ascent/atrophy and consequently an increased risk of infertility) than the development of a contralateral hernia would have. Especially in prematurely born children, who have the highest risk of developing an MCIH, contralateral exploration is very challenging because of their thin ‘premature’ structures that have a greater likelihood of being damaged. Even if the processus vaginalis is found to be open, the majority would still never develop into a clinically relevant hernia. On the other hand, MCIH development also carries risk for complications and often comprises multiple emergency department visits, e.g., due to children’s pain and discomfort and hernia incarceration (also with the subsequent risk for testicular atrophy). 

Anesthesiologists who were interviewed reported that one hour of anesthesia in children less than three years of age is considered safe. However, its influence on school performance and learning disabilities at a later age is still unclear. “*If it turns out that the total duration of exposure to anesthetics in the first year of life really matters*, *you can opt for one single prolonged session to limit the exposure*”—*Anesthesiologist*. However, anesthesiologists currently believe that a second short anesthesia of less than one hour does no further harm.

Parents of participants in the HERNIIA study stated that contralateral exploration itself is a relatively minor procedure that prevents second anesthesia and surgery and provides the assurance that a second hernia will not develop, even in case contralateral exploration was negative (i.e., the contralateral processus was found to be closed): “*It is actually only an extra incision*”—*parent of child in contralateral exploration group.* Parents of premature children especially preferred contralateral exploration because of the increased risk of MCIH development. Some of the parents of children with a difficult start after birth believed that the potential risks following contralateral exploration did not outweigh the risks during this rough period. They mentioned mostly relying on advice from an experienced surgeon, surgeons’ expert opinion thus acting as a facilitating factor. Others explicitly wanted to withhold their child from being exposed to these additional and potentially unnecessary complications just because they already had a lot to concur.

### 3.2. Theme 2: Stakeholders’ Motivation and Attitudes towards the Intervention

#### 3.2.1. Education and Standard of Care

All interviewed surgeons mentioned that unilateral hernia repair without contralateral exploration is currently considered the standard care for children with an inguinal hernia due to their past education and according to (local) protocols, which correspond to the current guidelines [7,13]. Education and standard of care thus both hinder contralateral exploration. “*In the field of pediatric surgery*, *you will learn from each other. Of course*, *all techniques are clearly described in books*, *though you mainly will learn it from your supervisor”—Pediatric surgeon.* Some centers/surgeons were or are still trained to discuss the opportunity to perform contralateral exploration with parents of children with certain comorbidities; however, *“The current guideline still states to only perform inguinal hernia repair in case a clinical hernia exists”—Pediatric surgeon and member of the guideline committee.*

#### 3.2.2. Expertise/Preference of Professionals

As discussed before, most interviewed surgeons mention that the small number of patients who develop an MCIH does not justify routine contralateral exploration. “*The less you have to operate the better. Here’s the trade-off that you have to perform an extra surgery without knowing whether the patient will actually benefit from it”—Pediatrician–neonatologist.* Both pediatricians and surgeons suggested that contralateral exploration to prevent second surgery might however be indicated (a) in case there is uncertainty about the presence of a contralateral hernia, (b) in patients with increased risk of developing an MCIH (e.g., premature or low-weight infants and children with a ventriculoperitoneal shunt in situ), or (c) in children with increased risk for developing complications during anesthesia (e.g., patients with pulmonary disease or premature infants who have a higher risk for apneas). 

### 3.3. Theme 3: Patient and Family Outcomes and Experience

#### 3.3.1. Informed Consent of Parents and Shared-Decision Making 

Informed consent of parents can act as a barrier and facilitating factor. Both parents and healthcare professionals reported that parents should be informed about both treatment options in case one is not more effective over the other. They should be able to choose whether they want their child to undergo contralateral exploration or not. “*I think it’s important that parents have a choice as there are potential complications”—parent of child in contralateral exploration group and parent of child in MCIH group.* Parents wanted to receive information on the success rates of the treatment, impact and risks of anesthesia and surgery (and not having surgery) specific for their child (taking into account his/her comorbidities), the potential negative effects of repetitive surgery and its timing, cosmetic aspects and long-term benefits and potential complications. Most interviewed parents preferred to receive both face-to-face and digital information to facilitate in the decision-making process. While some opt for shared decision making and value the surgeon’s expert opinion, others feel comfortable deciding on their own. “*Shared-decision making is of course very trendy these days*, *though requires parents to be well informed”—Pediatrician.*

#### 3.3.2. Experience of Parents 

Parents’ experience with previous hernia surgery increased the preference for contralateral exploration. The clinical manifestations of inguinal hernia, e.g., pain and discomfort of the child with sometimes inconsolable crying and lack of oral intake, together with multiple hospital visits, were especially considered very stressful by many parents. Consequently, the potential risks of contralateral exploration were by many parents considered less important than preventing this from happening a second time. “It was incredibly hard to see my child suffer. You just don’t want that to happen again”—Parent of child in contralateral exploration group. Parents of children who needed to undergo second surgery (i.e., the MCIH group) reported being more worried about the potential risks of repetitive anesthesia than they initially thought. Scar tissue after a potentially unnecessary incision withheld some parents from participation in the HERNIIA trial, though in hindsight: “If I had known that the postoperative scar was almost negligible, I might have chosen different”—Parent that declined participation in the HERNIIA trial. Multiple parents reported that they were surprised that their child recovered more quickly than they expected and without any complications.

### 3.4. Strategies to Overcome Barriers and Applicability in Hospital’s Organizational Structure 

Strategies that were mentioned to overcome the identified barriers were mainly related to support change and a multidisciplinary approach. 

#### 3.4.1. Support for Change

Support for changing the current treatment strategy will be dependent on the results of the HERNIIA study. Based on the currently available evidence, all interviewed surgeons were not very likely to perform contralateral exploration. They stated that inconclusive study results will also hinder the implementation of contralateral exploration and most surgeons then opt for de-implementation, earlier referred to as: *“In dubio abstine; i.e.*, *in case of doubt*, *don’t do”—Pediatric surgeon.* If the HERNIIA study reveals new evidence supporting the effectiveness of contralateral exploration: “I *think we can convince each other in a good discussion during one of the biannual meetings of the Dutch society of Pediatric Surgeons”—Pediatric surgeon.* (Pediatric) surgeons believed their close collaboration facilitates the implementation of new surgical treatments*. “I think education is a very strong tool to change things; you’re trained to be a surgeon at one place after which you will go work at another*, *where you will be an influencer”—Pediatric surgeon.* They suggested that new evidence either for or against contralateral exploration should be presented at (inter)national conferences, published in peer-reviewed journals and included in an updated version of the (inter)national guidelines on pediatric inguinal hernias to facilitate implementation. *“Following (inter)national implementation of the revised guideline it will automatically be included in the training program for new surgical residents”—Pediatric surgeon.* Operating room capacity is not expected to be a problem: “*It only creates more possibilities for training since there are more sides to operate on”—Pediatric surgeon.*

#### 3.4.2. Multidisciplinary Approach

According to all professionals that were interviewed, the surgeon should be the one to decide whether a child needs to undergo unilateral repair with or without contralateral exploration. Preoperatively, the surgeon can consult the anesthesiologist or (neonatologist) pediatrician to obtain patient-tailored advice on children with specific comorbidities who need to undergo surgery. “*You have to weigh a number of risks against each other and if you want to make a fair assessment of these risks*, *you must include the anesthetic risks as well”—Anesthesiologist.* Perioperatively, the anesthesiologist must also be able to advise the surgeon to (dis-)continue in case there are anesthetic problems or difficulties during surgery. 

## 4. Discussion

At the time of writing, the standard of care for children with a unilateral inguinal hernia includes open unilateral hernia repair [7,13]; there is no need to perform routine contralateral exploration [1,14,15,16,17,18,19]. Laparoscopic repair is increasingly popular, though it has not yet been embedded as a standard treatment for pediatric inguinal hernias. In the Netherlands, it is only recommended in children with an ipsilateral recurrent hernia [13]. In the US, only 9.3% of children younger than one year of age are treated with laparoscopic hernia repair [20]. The present study prospectively explored multiple stakeholders’ perceived factors that promote or hinder the potential adoption and implementation of contralateral groin exploration in children aged six months or younger undergoing unilateral hernia repair in the Netherlands. We identified three main themes that should be taken into account: (1) effectiveness of surgical treatment; (2) stakeholders’ motivation and attitudes towards the intervention; (3) patient and family outcomes and experience. Additionally, we pointed out strategies to overcome these barriers. Addressing these facilitating factors and potential barriers could help expedite implementation. 

Both healthcare professionals and parents identified the lack of decisive evidence as being one of the key barriers for the potential implementation of contralateral exploration. Also, the number needed to treat was considered too high to justify routine contralateral exploration, since over 80% of the patients with unilateral inguinal hernia will never develop a metachronous hernia. According to most interviewed healthcare physicians, the subsequent benefits of preventing second anesthesia and surgery in only the minority of patients did not outweigh the potential (unnecessary) complications. Parents who were not in favor of contralateral exploration also wanted to withhold their child from being exposed to additional (and potentially unnecessary) complications. Both these parents and healthcare professionals suggested that in case the risk of MCIH development in a specific group of children increases to, e.g., between 20 and 50%, routine contralateral exploration should be taken into reconsideration. Contrarily, many of the interviewed parents that preferred contralateral exploration experienced the clinical manifestations of an inguinal hernia as incredibly stressful and considered it a relatively minor procedure that prevents second anesthesia and surgery, as well as the convenience that a second hernia will not develop. 

In children aged six months or younger, the Dutch guidelines on pediatric inguinal hernias state that the use of contralateral exploration to prevent a MCIH, including its advantages and risks, should be discussed with their parents [13]. Peul et. al. previously discussed the importance of patients being well informed about both the surgical and conservative treatment strategy that aids in the decision-making process [21]. In line with this, we found that both parents and healthcare professionals reported that parents should be informed about both treatment options, mainly in case the results of the HERNIIA trial show that one treatment is not more effective over the other one. However, also in case one of the two treatments is shown to be more cost-effective or is associated with a lower complication rate, less parental stress or better quality of life for a specific (sub)group of patients, informed consent and shared decision making become increasingly important. What we have learned from the interviews in this qualitative study is that not only patient characteristics and the effectiveness of surgical treatment should be included in the process of decision making, but also parental distress and the quality of life of the child and their family. 

Parents who preferred contralateral exploration seemed to be less worried about potential complications of the intervention and emphasized the prevention of pain and discomfort because of the inguinal hernia of their child, together with the convenience that a second inguinal hernia will not develop. In view of that, previous studies showed that parents preferred inspection and repair of the contralateral region, if needed, more for convenience than for concerns about a second procedure or anesthesia [22,23]. They also showed that most parents preferred laparoscopic inspection over open exploration when presented with options regarding the management of the contralateral region in children with a unilateral hernia [22]. It is known that the use of laparoscopy to visualize and, if necessary, to repair the contralateral side results in a significantly lower rate of metachronous contralateral hernia repair [24]. However, laparoscopic repair has not yet been embedded as the standard treatment for Dutch children with primary inguinal hernia. Nonetheless, surgeons that were interviewed also believed that the introduction of laparoscopic inguinal hernia repair in children with a unilateral hernia would be a facilitator for the implementation of contralateral exploration. 

The HERNIIA trial will definitely provide new evidence on the effectiveness of contralateral groin exploration and its necessity in children with a unilateral inguinal hernia aged six months or younger, though the following question remains: will the evidence be enough? In the past, it has already been repeatedly concluded that routine contralateral exploration in children is not indicated, though both pediatricians and surgeons that were interviewed feel that contralateral exploration might still be indicated in children with an increased risk of developing a MCIH (e.g., premature or low-weight infants and children with a ventriculoperitoneal shunt in situ). Based on the currently available literature, most authors do not advocate routine exploration, though no clear recommendations can yet be made, and contralateral exploration in premature or low-weight infants might still be considered [15,25,26,27,28,29]. 

In case the results of the HERNIIA trial show that the superiority of the treatment strategies varies by outcome or by (sub)group of patients, interviewed healthcare professionals believe there will be a lot of support to change the standard of care. Shared decision making and, if relevant, the development of a decision tool would then be a suitable approach to decide which treatment best fits a child considering the best clinical evidence that balances risks and expected outcomes with patient preferences and values [30]. If the results are less decisive, most surgeons oppose against the introduction of a shared decision process and in that case even opt for complete de-implementation of contralateral exploration. All parents reported that, independent of the study results, they would prefer to receive information about both treatment options—with or without consultation of the treating surgeon—to be able to choose whether they want their child to undergo additional surgery or not. Over ten years ago, Nataraja et al. already suggested that in patients presenting with an originally left-sided hernia or who are less than six months old, a parental discussion should occur about the possible benefits and risks of contralateral exploration [1]. From the results of this study, it also became clear that not only professional but also individual beliefs and preferences of parents need to be taken into account when deciding between either treatment, as both treatment strategies have particular risks and benefits that might differ between patients. This will likely enhance implementation of contralateral exploration as some patients (e.g., with an initial left-sided inguinal hernia) are more likely to develop an MCIH, and thus will eventually benefit more from additional treatment [1,31]. 

### Strengths and Limitations

An important strength of this study is that all relevant stakeholders were included, and heterogeneity was ensured by including different healthcare professionals working at both university and non-university hospitals, stakeholders involved from a patients’ perspective and stakeholders at the organizational/policy level. We included both parents of children who participated in the HERNIIA trial (from the unilateral repair, the contralateral exploration and the MCIH group) and parents of children who rejected trial participation. For all stakeholder groups, data saturation was reached within the conducted number of interviews.

One of the limitations that should be addressed is that not all stakeholders that were considered to play a role in the care for children with an inguinal hernia (e.g., general practitioners, healthcare insurers) were included in this study. Socio-political factors are important for the (de-)implementation of treatment strategies, though these stakeholders are not involved in the treatment process. Financial compensation for both treatments was not an issue since both unilateral and bilateral inguinal hernia repair surgery in children is completely covered by insurance companies. (De-)implementation of contralateral exploration is not expected to change insurance coverage and thus the financial burden on the patient.

Since the present study was conducted in centers throughout the Netherlands, extrapolation of the results to other countries should be performed with caution. Another limitation includes that several forms of biases could be present due to the study design. Participant bias was limited by using a trained and independent ‘neutral’ moderator for the focus groups and by asking open-ended questions. The individual interviews were performed by two experienced researchers in qualitative interviewing. Coding was conducted individually by the same two researches to minimize researcher bias. 

## 5. Conclusions

Contralateral groin exploration versus no contralateral exploration in children with a unilateral inguinal hernia is considered a typical clinical equipoise. Based on currently available evidence, neither of the treatment strategies is expected to be revealed as the most (cost-)effective treatment strategy in infants aged six months or younger with a unilateral inguinal hernia. According to both healthcare professionals and parents of children with an inguinal hernia, the current number needed to treat is considered too high to justify routine contralateral exploration. In case the HERNIIA trial shows that one of the two treatments is more cost-effective or is associated with a lower complication rate, less parental stress or better quality of life for a specific (sub)group of patients, shared decision making with use of a decision aid would then be a suitable approach to decide which treatment best fits a child. We show that parental distress and the quality of life of the child and their family should be included in the process of decision making next to patient characteristics and the effectiveness of surgical treatment. In case the results of the trial are less decisive, parents will need a thorough understanding of the course of symptoms, complications and patient-reported outcomes to guide their decisions about both treatment strategies. 

## Figures and Tables

**Table 1:** Demographics of participating healthcare professionals.

	*N* = 9
Gender	
Male	5
Female	4
Occupation	
(Pediatric) surgeon	4
(Pediatric) surgery resident	1
Pediatrician	1
Pediatrician–neonatologist	1
Anesthesiologist	2
Years active in current role	
0–5	2
5–10	3
10–15	1
≥15	3
Type of organization	
University hospital	8
Non-university hospital	1

**Table 2:** Demographics of participating parents of children who underwent inguinal hernia repair.

	Individual Phone Calls with Non-Participating Parents in the HERNIIA Trial	Focus Groups (*N* = 2) with Participating Parents of the HERNIIA Trial
	*N* = 5	*N* = 12
Gender		
Male	1	3 ^a^
Female	4	9
Gender of child		
Male	4	8
Female	1	1
Treatment their child received		
Unilateral hernia repair (i.e., unilateral group)	2	2
Unilateral hernia repair + contralateral exploration (i.e., contralateral group)	3	5
Unilateral hernia repair and second surgery after development of MCIH (i.e., MCIH group)	-	2

^a^ Three parents participated together with their partner.

**Table 3:** Facilitators and barriers for implementation of contralateral exploration in the Netherlands.

Theme	Subtheme
1. Effectiveness of surgical treatment	1.1 Available evidence (−)1.2 Technical aspects of anesthesia and surgery (+/−)1.3 Complications of anesthesia and surgery (+/−)
2. Stakeholders motivation and attitudes towards the intervention	2.1 Education and standard of care (−)2.2 Expertise/preference of professionals (−)
3. Patient and family outcomes and experience	3.1 Informed consent of parents and shared decision making (+/−)3.2 Experience of parents (+/−)

+ = facilitating factor, − = hindering factor.

## Data Availability

The data presented in this study are available on request from the corresponding author.
